# Exploring the Prognostic Value and Immune Infiltration Patterns of GPRC5A Across Multiple Cancer Types

**DOI:** 10.7150/jca.93217

**Published:** 2024-07-09

**Authors:** Song Hu, Yan Chen, Beibei Sun

**Affiliations:** 1 Department of Clinical Laboratory Medicine, Shanghai Tenth People's Hospital of Tongji University School of Medicine, Shanghai, China.; 2 Institute for Thoracic Oncology, Shanghai Chest Hospital, School of Medicine, Shanghai Jiaotong University, Shanghai, China.

## Abstract

**Objective:** This study aimed to investigate the expression of GPRC5A in pan-cancer and its correlation with clinical outcomes, tumor immune microenvironment, and biological functions.

**Methods:** The expression of GPRC5A was analyzed using 33 tumor datasets from the TCGA, GTEx and TCGA databases. Immunohistochemical images from the HPA database were also examined. Kaplan-Meier survival analysis was conducted to assess the prognostic value of GPRC5A. Correlations between GPRC5A expression and clinical parameters were investigated. Nomogram models were developed to predict survival probabilities. The correlation between GPRC5A expression and tumor immune microenvironment was analyzed using the GEPIA2 database. Functional enrichment analysis and Gene Set Enrichment Analysis were performed to explore the biological functions associated with GPRC5A.

**Results:** GPRC5A exhibited varying expression levels across different types of tumors, with high expression observed in 11 types of cancer tissues. Aberrant GPRC5A expression was correlated with overall survival, disease-specific survival, and progression-free interval in specific cancers. Specific clinicopathological features were found to be associated with GPRC5A expression in six tumors. Nomogram models incorporating GPRC5A expression demonstrated significant clinical utility in predicting survival probabilities for patients with ACC, KIRC, LGG, and PAAD. GPRC5A was also found to be associated with the tumor immune microenvironment. Functional enrichment analysis revealed the involvement of GPRC5A-related genes in various biological processes and functions.

**Conclusion:** This study highlights the differential expression of GPRC5A in pan-cancer and its correlation with clinical outcomes. GPRC5A shows potential as a prognostic biomarker and therapeutic target in specific cancers. Moreover, its association with the tumor immune microenvironment suggests its involvement in the tumor immune response. The findings provide valuable insights into the biological roles of GPRC5A in tumors and contribute to our understanding of its clinical implications.

## Introduction

Cancer is one of the leading causes of death worldwide^1^, ^2^. According to Global Cancer Statistics, there were 19.29 million new cases of cancer and 9.96 million cancer-related deaths globally in 2020^3^. Due to factors such as aging populations and environmental pollution, it is projected that the burden of cancer will increase by 50% by 2040 compared to 2020, with nearly 30 million new cancer cases expected. Breast cancer, lung cancer, colorectal cancer, prostate cancer, stomach cancer, and liver cancer account for the majority of these deadly tumors.

In recent years, there has been notable progress made in the realm of treatment target research. Researchers are actively investigating key molecules within tumor signaling pathways with the aim of identifying potential therapeutic targets. Inhibitors that specifically target EGFR, HER2, ALK, and other molecular targets have shown remarkable therapeutic efficacy in select cancer types^4^-^6^. By analyzing the tumor genome, researchers can pinpoint mutations associated with tumor initiation and progression. An illustrative example is the exploration of BRAF V600E mutations in melanoma, which has led to the development and clinical application of BRAF inhibitors. In the field of cancer treatment, immune checkpoint inhibitors have emerged as a major breakthrough, with PD-1 and PD-L1 inhibitors exhibiting significant therapeutic effects across multiple cancer types. Nevertheless, challenges such as drug resistance and inter-individual differences persist. Therefore, there is an urgent need to find new methods for diagnosing and treating cancer, but our research progress has not been satisfactory. The lack of treatment targets is a problem that we urgently need to solve. We hope to find more suitable targets for the treatment of multiple tumors through the study of pan-cancer data.

G-protein-coupled receptor family C group 5 member A (GPRC5A), also known as retinoic acid-induced gene 1 (RAIG1) or retinoic acid-inducible gene 3 (RAI3), is localized on chromosome 12 (12p13.1) 7, 8. GPRC5A has emerged as a receptor of significant interest due to its complex involvement in cancer biology. Based on the current available data, GPRC5A has been implicated in the regulation of tumor initiation and progression. However, its role appears to be context-dependent, exhibiting contradictory effects in different types of tumors. It demonstrates tumor-suppressive functions in lung cancer^9^-^11^, while exerting pro-tumorigenic effects in breast cancer^12^, ^13^, pancreatic cancer^14^, ^15^ and colorectal cancer^16^, ^17^. The underlying molecular mechanisms and functional implications of GPRC5A remain poorly understood, and there is a lack of comprehensive pan-cancer analyses investigating its role.

In light of this, our research aims to investigate the involvement of GPRC5A in pan-cancer scenarios, with a particular focus on elucidating its interactions with the immune microenvironment. By unraveling the regulatory mechanisms of GPRC5A, I hope to gain insights into its potential as a therapeutic target across a variety of cancer types.

## Materials and Methods

### Data gathering and processing

The gene expression and clinical data of 33 tumors were collected from the Cancer Genome Atlas (TCGA)^18^. Immunohistochemical images of human normal tissues and tumor tissues were sourced from the Human Protein Atlas (HPA) website^19^. Additionally, we utilized the Gene Expression Profiling Interactive Analysis database (GEPIA2)^20^ to extract the 100 most relevant genes associated with GPRC5A from the TCGA datasets. This study was conducted in accordance with the guidelines provided by TCGA, thereby exempting the need for ethical approval and patient consent.

### Bioinformatics analysis of GPRC5A expression levels in cancers

The mRNA expression of GPRC5A in normal tissues and tumor tissues was compared separately in TCGA_GTEx samples, TCGA samples, and TCGA paired samples. Furthermore, the protein level of GPRC5A in both normal and tumor tissues was investigated by using the HPA database.

### Correlation analysis between GPRC5A expression and clinicopathological characteristics of cancer patients

The association between clinical outcomes including overall survival (OS), progression-free interval (PFI), disease-specific survival (DSS), gender, T stage, N stage and pathologic stage in pan cancer from TCGA and GPRC5A expression was assessed using Kaplan-Meier analysis with log-rank test. The survival curves with p-values less than 0.05 were shown. Furthermore, receiver operating characteristic (ROC) curves were drawn in tumors where prognosis can be influenced by GPRC5A.

### Establishment and assessment of the nomogram models

In the present study, a univariate Cox regression analysis was conducted for overall survival (OS) in tumors where GPRC5A can impact prognostic outcomes, including OS, PFI, and DSS. Tumors with a significance level (p-value) of less than 0.05 and a sample size greater than 500 were chosen to establish separate nomogram models. The calibration curves were performed to assess the prediction accuracy of the nomograms at 1-year, 3-year, and 5-year. These data were analyzed using the “survival” (v3.3.1) R package (statistical analysis of survival data) and “rms” (6.3-0) R package (visualization) for the prognostic analysis.

### Immune infiltration analysis

The correlations between GPRC5A expression and various immune cell types, such as B cells, macrophages, CD4+ T cells, and CD8+ T cells, were analyzed using the Tumor Immune Estimation Resource 2.0 (TIMER2.0)^21^ across all tumors in TCGA. Furthermore, we investigated the impact of immune cell infiltration on overall survival (OS) by stratifying GPRC5A expression in different tumor types.

### Functional enrichment analysis of GPRC5A-associated Differentially Expressed Genes in pan cancer

The GEPIA2 database was utilized to identify the top 100 genes that display a similar expression pattern to GPRC5A. These genes were then subjected to gene ontology (GO) analysis, which includes biological pathways (BP), cellular components (CC), and molecular functions (MF). Additionally, Kyoto Encyclopedia of Genes and Genomes (KEGG) analysis was conducted to further investigate the potential functions of GPRC5A.

To examine the differential expression of GPRC5A in cancers where it can impact prognosis, the DESeq R package was employed. Subsequently, based on the results obtained from the differential expression analysis of GPRC5A in different tumor types, Gene Set Enrichment Analysis (GSEA) was carried out using the cluster Profiler R package (v3.14.3).

### Statistical analysis

The Wilcoxon rank-sum test was utilized to compare the differences between two groups, while the correlation between two groups was evaluated using the Spearman rank test. Univariate and multivariate Cox proportional hazard regression analyses were performed to identify factors that influence prognosis. Kaplan-Meier analysis with the log-rank test was employed for survival analysis. The data were presented as mean ± standard deviation. The statistical analysis was conducted using R software (version 4.0.2), and a p-value of less than 0.05 was considered statistically significant (*p < 0.05, **p < 0.01, ***p < 0.001).

## Results

### The expression of GPRC5A in pan-cancer

**Figure 1:**
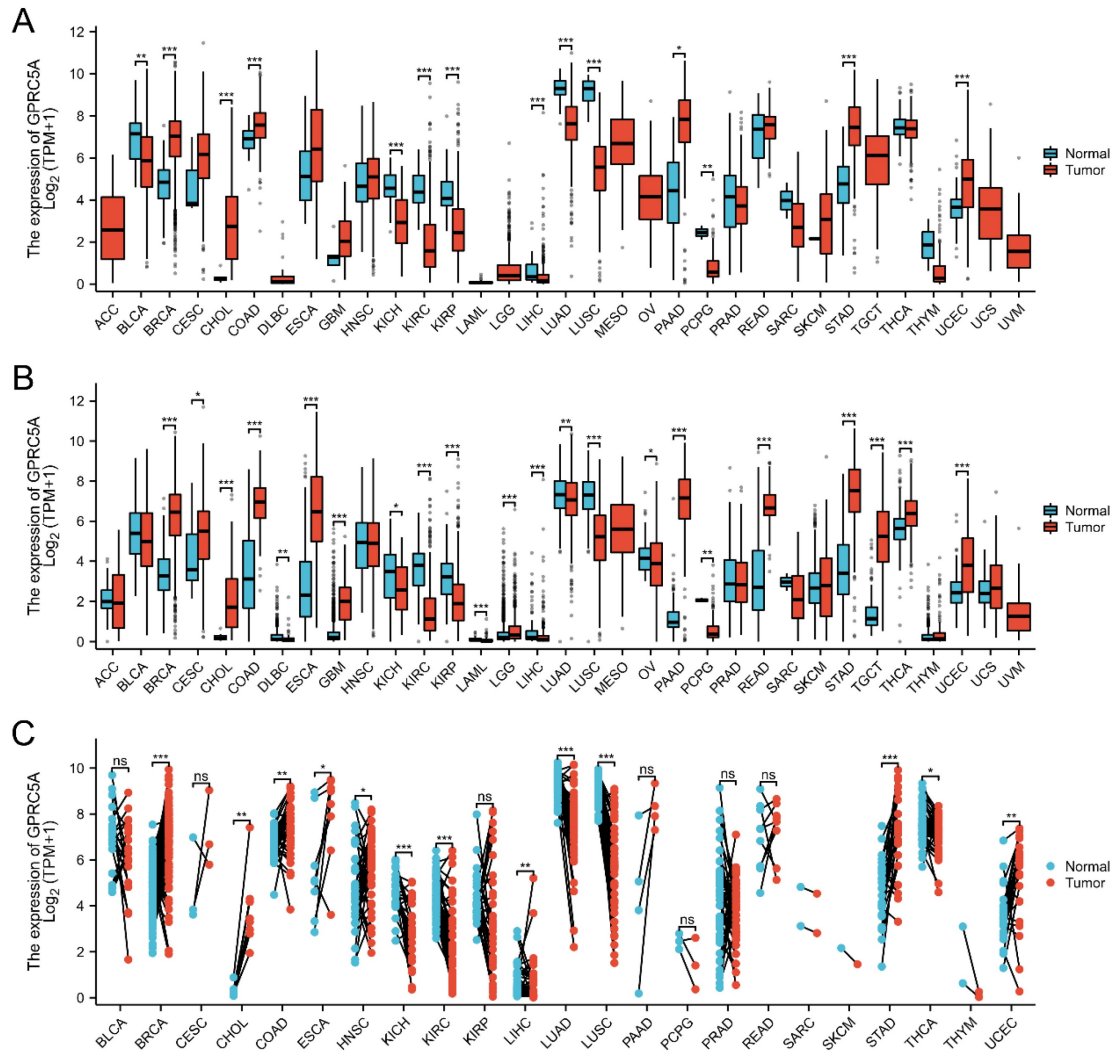
The mRNA expression of GPRC5A in pan-cancer. (A) and (B) The expression of GPRC5A mRNA across 33 types of tumors was analyzed in TCGA_GTEx and TCGA databases; (C) Expression of GPRC5A in paired samples of 23 tumors in TCGA database. (ns, p > 0.05; *p < 0.05; **p < 0.01; ***p < 0.001).

GPRC5A expression was analyzed in 33 tumor datasets from the TCGA_GTEx and TCGA database. The results indicated that GPRC5A exhibited varying expression levels across different types of tumors. Among them, 11 types of cancer tissues showed high expression, while others displayed low expression or no significant difference. (Figure 1A). These observations generally aligned with the results obtained from the TCGA dataset (see Figure 1B). Furthermore, the expression of GPRC5A was examined in 23 types of tumors using paired samples from the TCGA database (Figure 1C).

**Figure 2:**
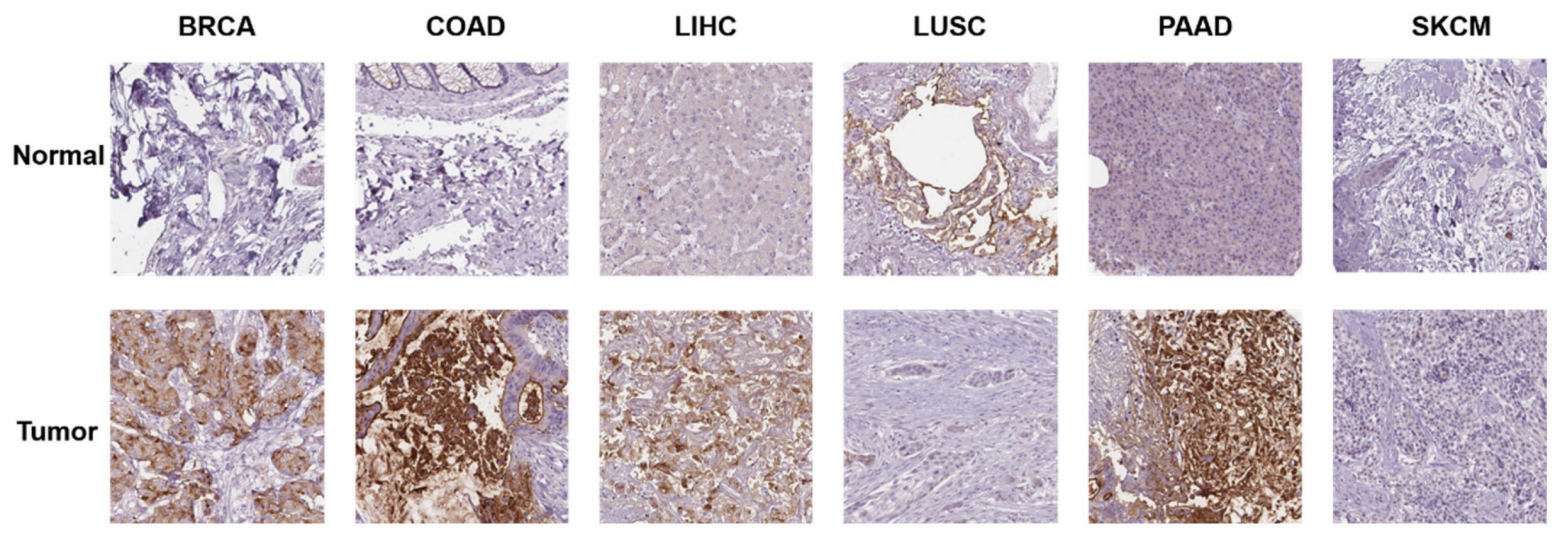
The protein expression of GPRC5A in normal and tumor tissues.

Additionally, we investigated the protein expression of GPRC5A in normal and tumor tissues from various human organs using the HPA database. Representative immunohistochemical (IHC) images of both normal and tumor tissues were extracted for analysis. The data indicates that GPRC5A is overexpressed in breast cancer, colorectal cancer, hepatocellular carcinoma, and pancreatic cancer, while it is underexpressed in lung squamous cell carcinoma. There is no significant difference in its expression in melanoma. Overall, these findings are consistent with the mRNA detection data from TCGA (Figure 2).

### The correlation between the expression of GPRC5A and prognosis in pan-cancer

**Figure 3:**
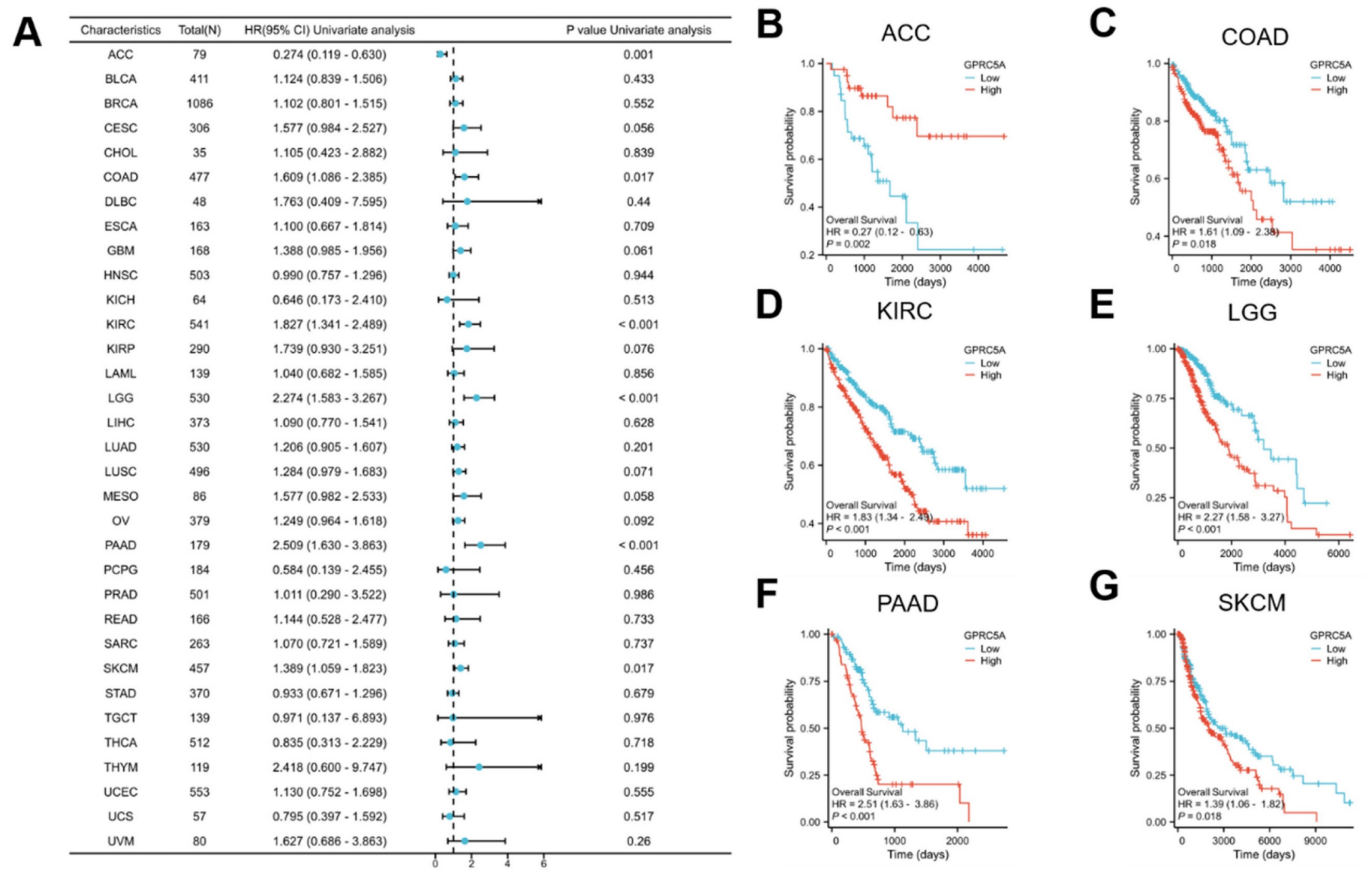
The correlation between the expression of GPRC5A and OS in pan-cancer. (A) The impact of GPRC5A expression on OS in pan-cancer was exhibited by a forest plot; (B-G) The influence of GPRC5A expression on OS in ACC, COAD, KIRC, LGG, PAAD and SKCM was individually assessed.

To assess the prognostic value of GPRC5A in pan-cancer, we conducted Kaplan-Meier survival analysis to examine the relationship between GPRC5A expression and clinical outcomes. Initially, we investigated the association between GPRC5A expression and overall survival (OS) in 33 different cancers (Figure 3A). The results revealed that aberrant GPRC5A expression was correlated with OS in several specific cancers: ACC (Figure 3B), COAD (Figure 3C), KIRC (Figure 3D), LGG (Figure 3E), PAAD (Figure 3F), and SKCM (Figure 3G). High expression of GPRC5A was associated with longer OS in ACC, while high expression of GPRC5A in COAD, LGG, PAAD, and SKCM was linked to shorter OS.

**Figure 4:**
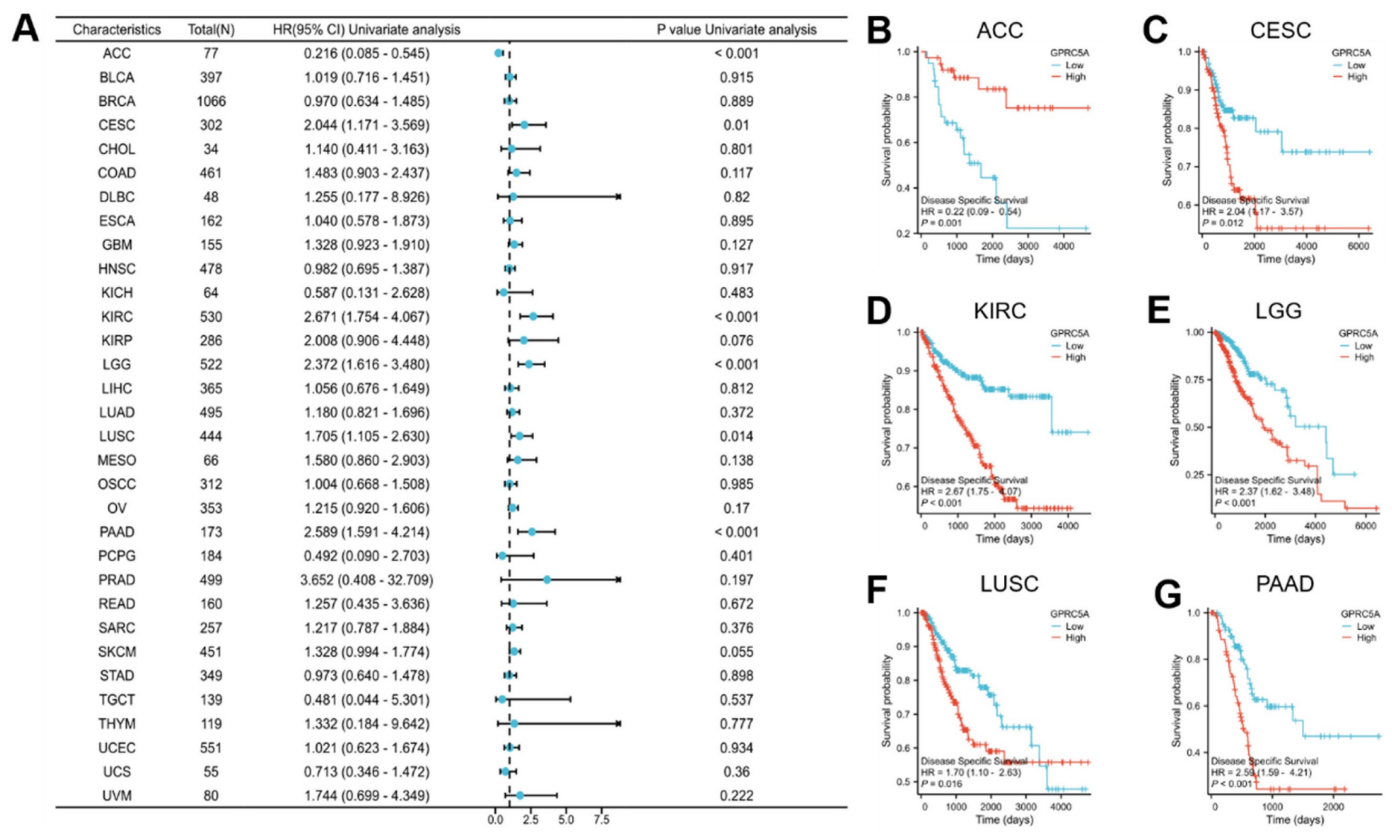
The relationship between the expression of GPRC5A and DSS in pan-cancer. (A) The impact of GPRC5A expression on DSS in pan-cancer was exhibited by a forest plot; (B-G) The effect of GPRC5A expression on DSS in ACC, CESC, KIRC, LGG, LUSC and PAAD was individually examined.

Subsequently, we explored the association between GPRC5A expression and disease-specific survival (DSS) (Figure 4A), revealing a correlation between GPRC5A expression and DSS in ACC (Figure 4B), CESC (Figure 4C), KIRC (Figure 4D), LGG (Figure 4E), LUSC (Figure 4F), and PAAD (Figure 4G). In ACC, higher expression of GPRC5A was linked to improved DSS, whereas in CESC, KRIC, LGG, LUSC, and PAAD, higher expression of GPRC5A indicated worse DSS.

**Figure 5:**
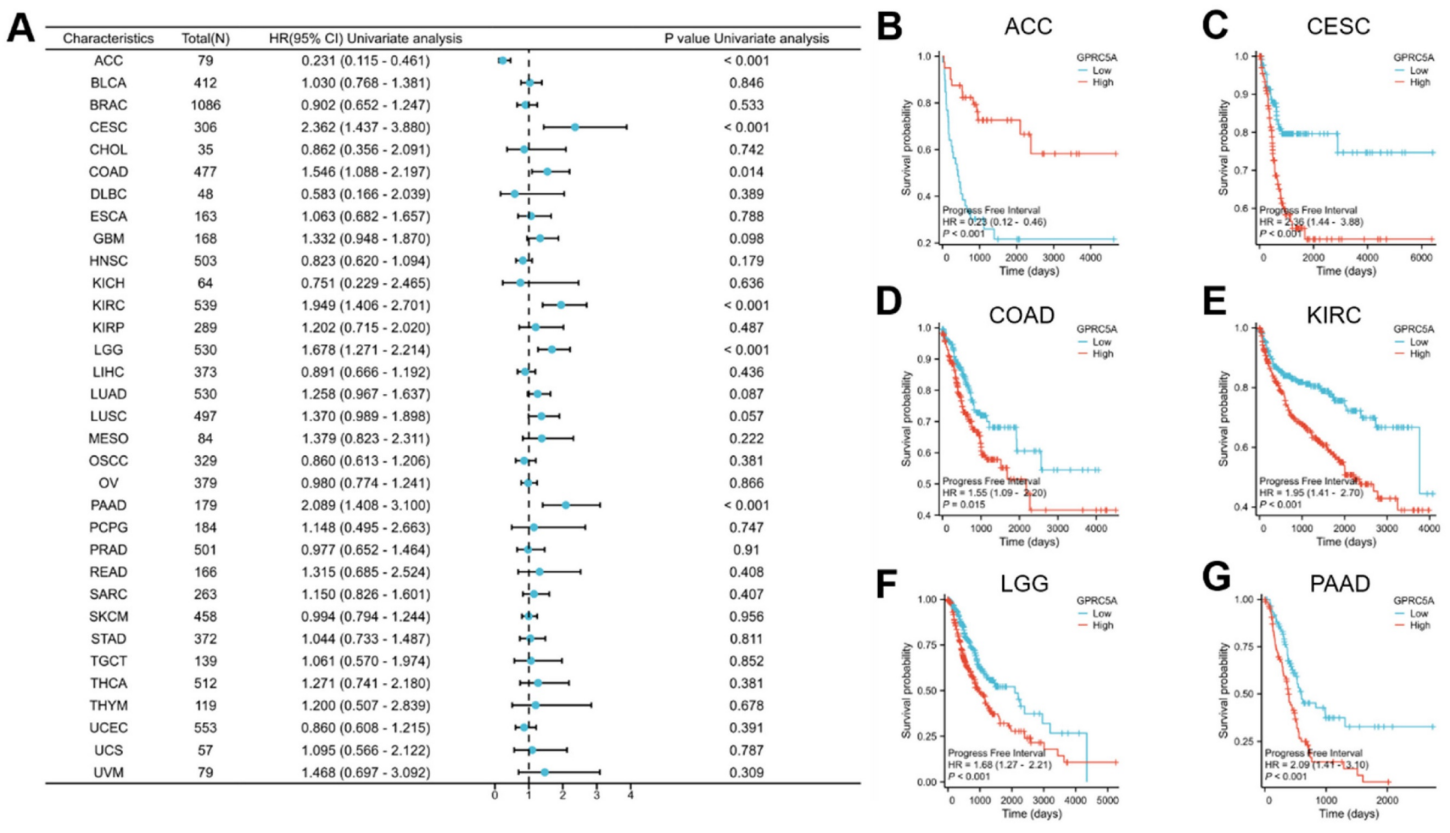
The association between the expression of GPRC5A and PFI in pan-cancer. (A) The impact of GPRC5A expression on PFI in pan-cancer was exhibited by a forest plot; (B-G) The effect of GPRC5A expression on PFI in ACC, CESC, KIRC, LGG, LUSC and PAAD was individually evaluated.

Lastly, we investigated the association between GPRC5A expression and progression-free interval (PFI) (Figure 5A). The results revealed a correlation between GPRC5A expression and PFI in ACC (Figure 5B), CESC (Figure 5C), COAD (Figure 5D), KIRC (Figure 5E), LGG (Figure 5F), and PAAD (Figure 5G). Higher expression of GPRC5A was associated with worse PFI in CESC, COAD, KIRC, LGG, and PAAD, but conversely, it indicated better PFI in ACC.

### The correlations the expression of GPRC5A and clinical parameters in pan-cancer

**Figure 6:**
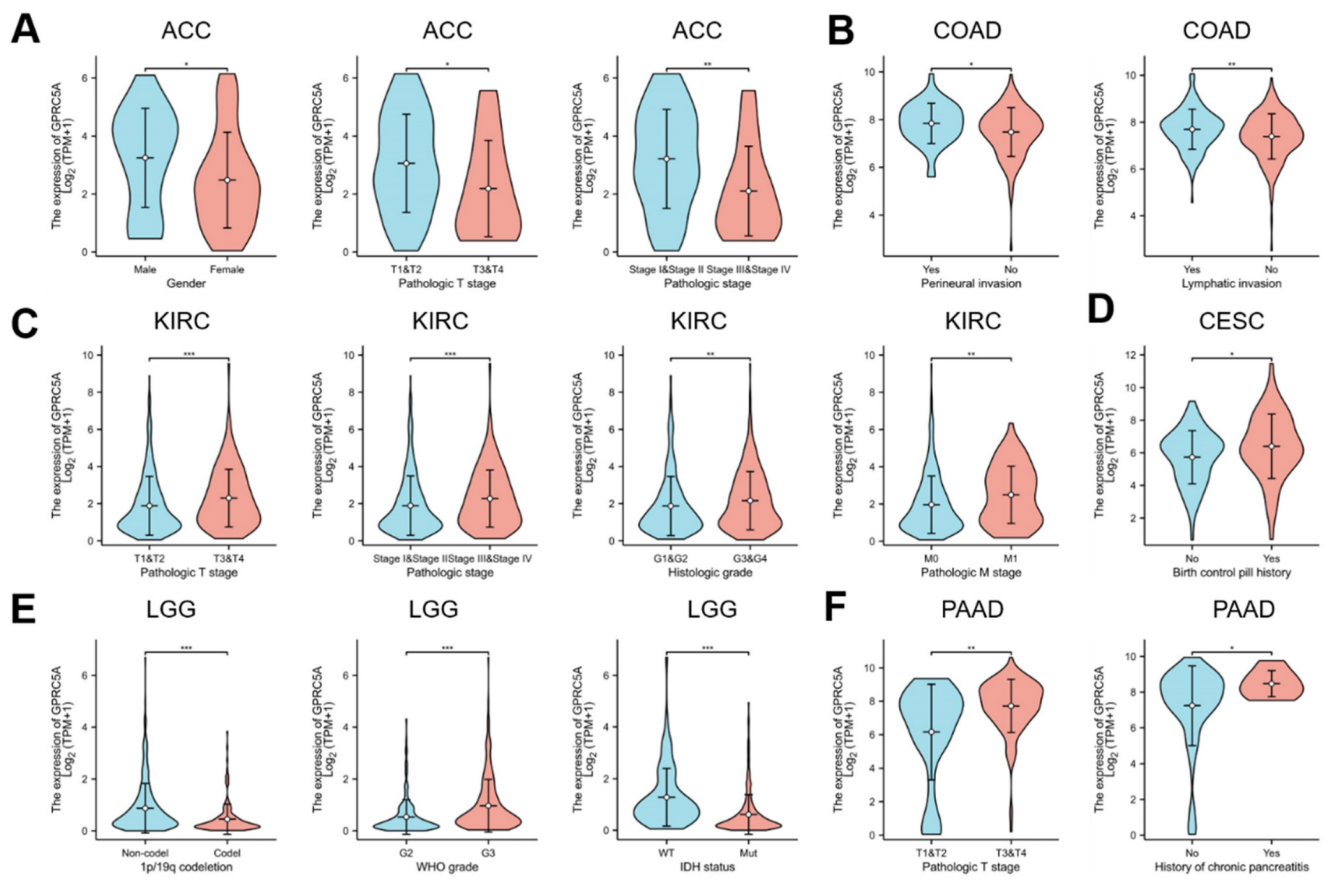
The correlation between GPRC5A expression and clinicopathological parameters. (A) GPRC5A expression was correlated with gender, Gender, Pathological T stage, and Pathological stage in ACC;(B) GPRC5A expression was correlated with Perineural invasion and Lymphatic invasion in COAD; (C) GPRC5A expression was associated with Pathological T stage, Pathological stage, Histologic grade, and Pathological M stage in KIRC;(D) GPRC5A expression was associated with a history of using birth control pills in CESC; (E) GPRC5A expression was correlated with 1p/19q codeletion, WHO grade, and IDH status in LGG; (F) GPRC5A expression in PAAD was correlated with Pathological T stage and a history of chronic pancreatitis. (ns, p > 0.05; *p < 0.05; **p < 0.01; ***p < 0.001).

Our analysis revealed that the expression of GPRC5A was associated with prognosis in six tumors from the TCGA database: ACC, COAD, KIRC, CESC, LGG, and PAAD. Further investigation of these tumors revealed specific clinicopathological features that correlated with GPRC5A expression. In ACC, GPRC5A expression was related to Gender, Pathological T stage, and Pathological stage (Figure 6A). The expression of GPRC5A in COAD was correlated with Perineural invasion and Lymphatic invasion (Figure 6B). In KIRC, GPRC5A expression was associated with Pathological T stage, Pathological stage, Histologic grade, and Pathological M stage (Figure 6C). CESC showed an association between GPRC5A expression and a history of using birth control pills (Figure 6D). In LGG, GPRC5A expression was found to be linked to 1p/19q codeletion, WHO grade, and IDH status (Figure 6E). Lastly, GPRC5A expression in PAAD was correlated with Pathological T stage and a history of chronic pancreatitis (Figure 6F).

### Developing and assessing nomogram models across four tumor types

**Figure 7:**
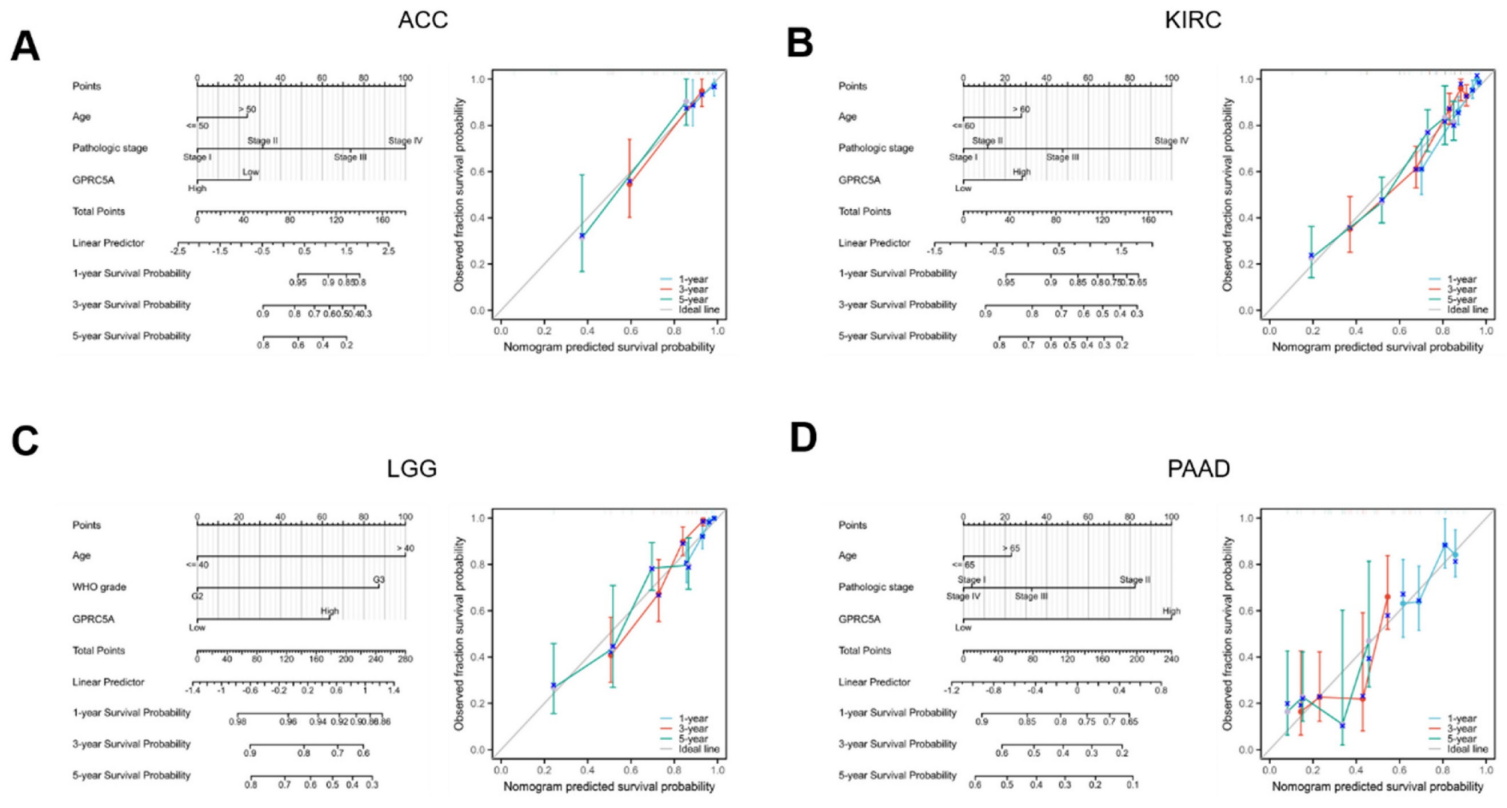
Establishment and evaluation of nomogram models in ACC, KIRC, LGG, and PAAD. (A-D) Establishment of a nomogram model incorporating GPRC5A expression and calibration curves in ACC, KIRC, LGG and PAAD at 1-year, 3-year, and 5-year.

To explore the impact of GPRC5A expression on prognosis in specific cancers, we conducted univariate Cox regression analysis for overall survival in four tumors where GPRC5A had been previously linked to prognosis. Using the results of this analysis, we developed nomogram models that incorporated T stages, tumor status, pathologic stages, and GPRC5A expression levels as predictors. These factors were identified as highly significant prognostic predictors through multivariate Cox regression analysis. The nomogram demonstrated significant clinical utility in predicting 1-, 3-, and 5-year survival probabilities for patients with ACC (Figure 7A), KIRC (Figure 7B), LGG (Figure 7C) and PAAD (Figure 7D).

### The correlation between the expression of GPRC5A and tumor immune microenvironment in pan-cancer

**Figure 8:**
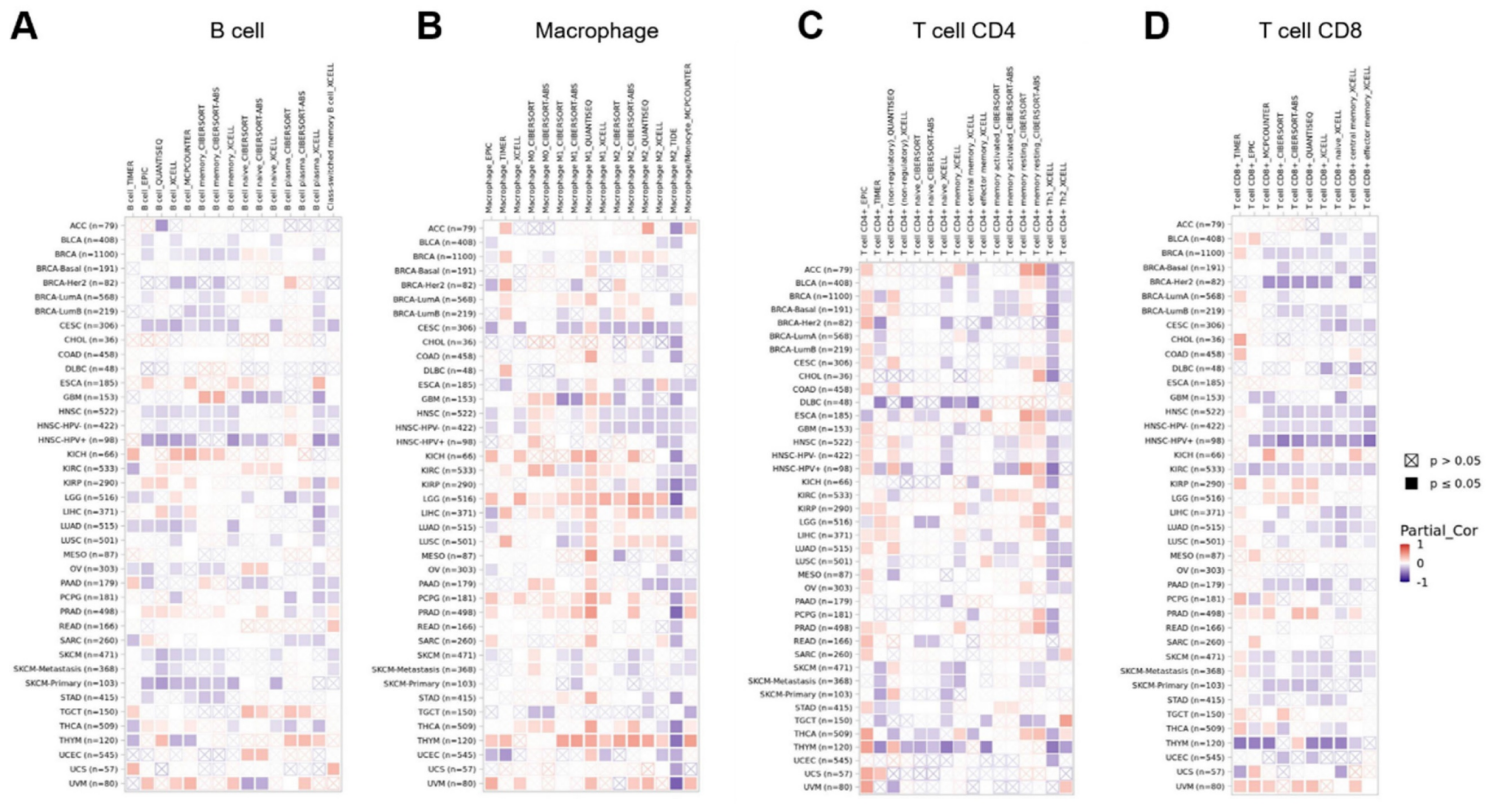
The correlation between the expression of GPRC5A and tumor immune microenvironment. (A-D) Heatmaps illustrating the correlations between GPRC5A expression and immune cell types including B cells, macrophages, T cell CD4+, and T cell CD8+ in the TIMER2 database, respectively.

The immune microenvironment has a critical role in the initiation and progression of tumors. To explore the link between GPRC5A and tumor immune microenvironment across multiple cancer types, we analyzed the correlation between GPRC5A expression and immune cells using the GEPIA2 database. Heatmaps were generated to visualize the correlation between GPRC5A expression and B cells (Figure 8A), macrophages (Figure 8B), CD4+ T cells (Figure 8C), and CD8+ T cells (Figure 8D) in pan-cancer.

### Functional enrichment analysis and Gene Set Enrichment Analysis

**Figure 9:**
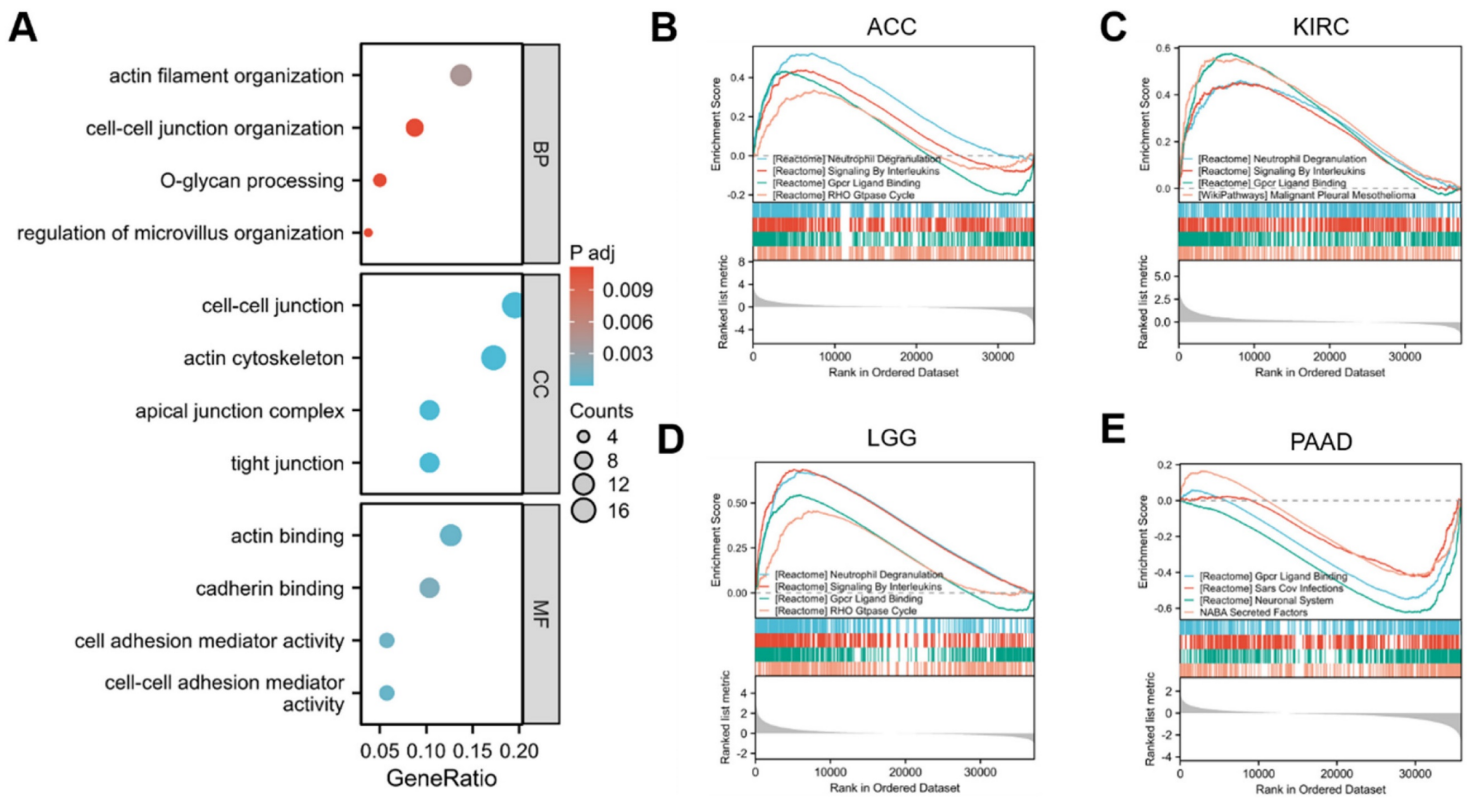
Functional enrichment analysis and Gene set enrichment analysis of genes correlated with GPRC5A. (A) GO enrichment analysis based on 100 GPRC5A-related genes, including BP, CC, and MF. (B-E) GSEA analysis was conducted based on the analysis of differential GPRC5A expression in ACC, KIRC, LGG and PAAD, respectively.

In order to gain deeper insights into the biological role of GPRC5A in tumors, we retrieved the top 100 genes most correlated with GPRC5A from the GEPIA2 database. The gene ontology (GO) analysis indicated that genes related to GPRC5A may be involved in various biological processes such as "actin filament organization," "cell-cell junction organization," "O-glycan processing," "regulation of microvillus organization," "actin binding," and "cadherin binding" (Figure 9A).

To further investigate the functional implications of GPRC5A, we performed Gene Set Enrichment Analysis (GSEA) based on differential expression analysis of GPRC5A. This analysis aimed to elucidate the biological functions associated with GPRC5A in four tumors where its expression was prognostically significant: ACC (Figure 9B), KIRC (Figure 9C), LGG (Figure 9D), and PAAD (Figure 9E). The findings revealed that GPRC5A was primarily associated with neutrophil degranulation, signaling by interleukins, GPCR ligand binding, and the RHO GTPase cycle.

In summary, GPRC5A is overexpressed in multiple cancer types and promotes cancer development and progression through various mechanisms, including the regulation of immune cell infiltration into tumors. Our study demonstrates that GPRC5A represents a promising prognostic and immunotherapeutic biomarker in certain malignant tumors.

## Discussion

Tumors pose a serious threat to human health, and research has shown that the earlier they are detected, the better the chances of survival^22^, ^23^. Despite the discovery of many biomarkers, many tumors still lack effective identification markers and treatment targets. Therefore, mining tumor biomarkers from existing massive data holds great significance.

This study aimed to investigate the expression of GPRC5A in pan-cancer and its correlation with clinical outcomes, tumor immune microenvironment, and biological functions. The results showed that GPRC5A exhibited high expression in 11 types of cancer tissues, while it was underexpressed or showed no significant difference in others. Aberrant GPRC5A expression was correlated with overall survival, disease-specific survival, and progression-free interval in several specific cancers. Furthermore, GPRC5A expression was associated with specific clinicopathological features in six tumors. Nomogram models incorporating GPRC5A expression demonstrated significant clinical utility in predicting survival probabilities for patients with ACC, KIRC, LGG, and PAAD. GPRC5A was also found to be linked to the tumor immune microenvironment across multiple cancer types. Functional enrichment analysis and Gene Set Enrichment Analysis revealed that GPRC5A may be involved in various biological processes and functions, including those related to cell junction organization and actin binding. Overall, this study provides insights into the expression and biological roles of GPRC5A in tumors and highlights its potential as a biomarker for prognosis and therapeutic target in specific cancers.

We are interested in the fact that GPRC5A shows different expression levels in various types of tumors, but its impact on tumor development is contradictory. GPRC5A is overexpressed in normal lung cells and has been widely proven to be a lung cancer suppressor gene. Additionally, GPRC5A-deficient mouse models are considered to be the closest representation of spontaneous human lung cancer^24^, ^25^. Surprisingly, in this study, we did not find significant differences in the expression level or functional effect of the GPRC5A gene in lung cancer, suggesting that its regulation in lung cancer primarily occurs at the post-transcriptional level. Our research data show that GPRC5A is overexpressed in other tumors such as pancreatic cancer, breast cancer, and colon cancer, and is negatively correlated with patient survival, consistent with existing research findings, highlighting the complexity of its intrinsic regulatory mechanisms. Furthermore, the data shows that GPRC5A is downregulated in LUSC and KIRC, but it is negatively correlated with patient survival and tumor progression. These findings suggest the complexity of tumor gene research, which may be related to genetic mutations, post-transcriptional regulation of expression, or epigenetic modifications of GPRC5A.Therefore, further understanding of the regulatory mechanisms of GPRC5A is not only beneficial for identifying tumor markers but also provides reliable targets for cancer treatment.

We have several advantages in our study. First, we explored the expression and regulation of GPRC5A in a wide range of cancers, including analyses of expression differences, clinical correlations, survival rates, and immune infiltration. This deepened our understanding of the function of the GPRC5A gene in tumorigenesis. Second, in 33 different types of tumors, we found that the co-expressed genes of GPRC5A are closely related to critical signaling pathways, such as neutrophil degranulation, signaling by interleukins, GPCR ligand binding, and the RHO GTPase cycle, providing new insights into its regulatory mechanisms. Although we conducted detailed analysis on GPRC5A data, there are still limitations, such as small sample size and lack of in vitro and in vivo experimental validation.

In conclusion, our study sheds light on the intricate and multifaceted nature of GPRC5A's involvement in tumorigenesis across different cancer types. Further research aimed at unraveling the specific regulatory mechanisms of GPRC5A will not only enhance our understanding of tumor biology but also provide valuable opportunities for the development of targeted cancer therapies.

## References

[1] Hanahan D (2022). Hallmarks of Cancer: New Dimensions. Cancer Discov.

[2] Ruiz-Casado A, Martin-Ruiz A, Perez LM, Provencio M, Fiuza-Luces C, Lucia A (2017). Exercise and the Hallmarks of Cancer. Trends Cancer.

[3] Sung H, Ferlay J, Siegel RL, Laversanne M, Soerjomataram I, Jemal A (2021). Global Cancer Statistics 2020: GLOBOCAN Estimates of Incidence and Mortality Worldwide for 36 Cancers in 185 Countries. CA Cancer J Clin.

[4] Wu SG, Shih JY (2018). Management of acquired resistance to EGFR TKI-targeted therapy in advanced non-small cell lung cancer. Mol Cancer.

[5] Han H, Li S, Chen T, Fitzgerald M, Liu S, Peng C (2021). Targeting HER2 Exon 20 Insertion-Mutant Lung Adenocarcinoma with a Novel Tyrosine Kinase Inhibitor Mobocertinib. Cancer Res.

[6] Cicin I, Martin C, Haddad CK, Kim SW, Smolin A, Abdillah A (2022). ALK TKI therapy in patients with ALK-positive non-small cell lung cancer and brain metastases: A review of the literature and local experiences. Crit Rev Oncol Hematol.

[7] Cheng Y, Lotan R (1998). Molecular cloning and characterization of a novel retinoic acid-inducible gene that encodes a putative G protein-coupled receptor. J Biol Chem.

[8] Acquafreda T, Soprano KJ, Soprano DR (2009). GPRC5A: A potential tumor suppressor and oncogene. Cancer Biol Ther.

[9] Lin X, Zhong S, Ye X, Liao Y, Yao F, Yang X (2014). EGFR phosphorylates and inhibits lung tumor suppressor GPRC5A in lung cancer. Mol Cancer.

[10] Song H, Ye X, Liao Y, Zhang S, Xu D, Zhong S (2023). NF-kappaB represses retinoic acid receptor-mediated GPRC5A transactivation in lung epithelial cells to promote neoplasia. JCI Insight.

[11] Wang T, Jing B, Xu D, Liao Y, Song H, Sun B (2020). PTGES/PGE(2) signaling links immunosuppression and lung metastasis in Gprc5a-knockout mouse model. Oncogene.

[12] Sokolenko AP, Bulanova DR, Iyevleva AG, Aleksakhina SN, Preobrazhenskaya EV, Ivantsov AO (2014). High prevalence of GPRC5A germline mutations in BRCA1-mutant breast cancer patients. Int J Cancer.

[13] Klaschik K, Hauke J, Neidhardt G, Trankle C, Surowy HM, Heilmann-Heimbach S (2019). The GPRC5A frameshift variant c.183del is not associated with increased breast cancer risk in BRCA1 mutation carriers. Int J Cancer.

[14] Zhou H, Telonis AG, Jing Y, Xia NL, Biederman L, Jimbo M (2016). GPRC5A is a potential oncogene in pancreatic ductal adenocarcinoma cells that is upregulated by gemcitabine with help from HuR. Cell Death Dis.

[15] Liu B, Yang H, Pilarsky C, Weber GF (2018). The Effect of GPRC5a on the Proliferation, Migration Ability, Chemotherapy Resistance, and Phosphorylation of GSK-3beta in Pancreatic Cancer. Int J Mol Sci.

[16] Greenhough A, Bagley C, Heesom KJ, Gurevich DB, Gay D, Bond M (2018). Cancer cell adaptation to hypoxia involves a HIF-GPRC5A-YAP axis. EMBO Mol Med.

[17] Zhang L, Li L, Gao G, Wei G, Zheng Y, Wang C (2017). Elevation of GPRC5A expression in colorectal cancer promotes tumor progression through VNN-1 induced oxidative stress. Int J Cancer.

[18] Blum A, Wang P, Zenklusen JC (2018). SnapShot: TCGA-Analyzed Tumors. Cell.

[19] Uhlen M, Fagerberg L, Hallstrom BM, Lindskog C, Oksvold P, Mardinoglu A (2015). Proteomics. Tissue-based map of the human proteome. Science.

[20] Tang Z, Kang B, Li C, Chen T, Zhang Z (2019). GEPIA2: an enhanced web server for large-scale expression profiling and interactive analysis. Nucleic Acids Res.

[21] Li T, Fu J, Zeng Z, Cohen D, Li J, Chen Q (2020). TIMER2.0 for analysis of tumor-infiltrating immune cells. Nucleic Acids Res.

[22] Kalluri R, McAndrews KM (2023). The role of extracellular vesicles in cancer. Cell.

[23] Davies MPA, Sato T, Ashoor H, Hou L, Liloglou T, Yang R (2023). Plasma protein biomarkers for early prediction of lung cancer. EBioMedicine.

[24] Chen Y, Deng J, Fujimoto J, Kadara H, Men T, Lotan D (2010). Gprc5a deletion enhances the transformed phenotype in normal and malignant lung epithelial cells by eliciting persistent Stat3 signaling induced by autocrine leukemia inhibitory factor. Cancer Res.

[25] Tao Q, Fujimoto J, Men T, Ye X, Deng J, Lacroix L (2007). Identification of the retinoic acid-inducible Gprc5a as a new lung tumor suppressor gene. J Natl Cancer Inst.

